# 

**Published:** 1840-01-04

**Authors:** 


### CONTENTS OF VOL. III.

A

Abortion, cases of 185, 432.
Albumen in dysentery, 368.
Allison, death of Dr., 205.
Almanac, American Medical, 80, 795.
Amputation, statistics of, 298; Dr. Horner's case of at the shoulder-joint, 549; of the penis, 628.
Anasarca after Scarlatina, 193.
Aneurism of the Aorta, Dr. Pennock's case of, 73; case of, cured by acetate of lead, 175; cases of, 221, 227, 367, 474, 777.
Antimonial vapours, poisoning from, 739.
Aorta, cases of acute inflammation of the, 658.
Archives de Médécine, notice of, 277.
Arsenic, cases of poisoning cured by hydrated peroxide of iron, 250, 679; cases of poisoning from, 433, 468.
Artery-needle, Dr. Horner's new, 549.
Arthritis, on, 580.
Association, British, transactions of the, 676, 803.
Asthma, lecture on, 126.
Authors and reviewers, 807.

B

Baryta, hydrochlorate of, in ophthalmia, 368.
Bladder, on the puncture of the, 527.
Blephorastic operations, 384.
Blood, M. Andral on alterations of the, 396, 414, 456, 565, 599.
Blood-vessels, on the contractility of the, 728.
Brainard, professor, introductory lecture by, 115.
Brigham on diseases of the nervous system, notice of, 248.
Burns, post-mortem appearances after, 492.

C

Cæsarean operation, history of, in a twin case, 386.
Calculus, in the urethra, 622.
Calomel with iodine and sugar, 447.
Cancer, M. Andral on, 317; new treatment of, 436; on the formation of in the veins, 461.
Capillary circulation, recent experiments on, 281.
Cervix uteri, on ulcerations of, 131; amputation of, 260.
Cheese, case of poisoning from, 386.
Chest, lectures on the exploration and treatment of diseases of, 309, 325, 341, 357, 373, 389, 405, 421, 437, 533, 581, 613, 661, 757, 821.
Chlorosis, lactate of iron in, 526.
Chorea, ferri ferrocyanus in, 314.
Clavicle, extirpation of, 385, 623, 691.
Club-foot, Dr. Detmold on, 127; cases of, 164.
Cœcum, diseases of the, 580.
Colchicum seeds, poisoning from, 527.
Colleges, Philadelphia Medical, 45; Louisville Medical Institute, 115, 454; Transylvania, 205; University of Pennsylvania, 216; Pennsylvania Medical, 216, 454; Albany Medical, 216; Geneva, 454; Vermont, 648; Yale, 694.
Colocynth oil, a substitute for croton, 660.
Compound fractures, cases of, 202.
Conception after cessation of the menses, 196.
Concours for the chair of internal pathology in Paris, 393, 425.
Concretions, sanguineous, 430.
Convention, National Medical, proceedings of, 58.
Convulsions, puerperal, case of, 427.
Copaiva, formula for, 131.
Copper, mode of detecting poisoning by, 563.
Cornea, on the nerves of, 451, 468.
Cotton-plant, medical properties of, 553.
Creosote in deafness, Dr. Partridge on, 347.
Croup, on the pathology and treatment of, 625.
Cystocele, operation for the cure of vaginal, 652.

D

Danish medical statistics, 756.
Delirium tremens, lectures on, 46.
Dextrine and diastase, Mr. Procter on, 52, 65, 81.
Diaphragm, congenital, hernia of, 384.

Dictionnaire de Médécine, notices of, 247, 277.
Diospyros Virginiana, case of poisoning from, 198.
Dislocations, of the cervical vertebræ, case of, 120; of humerus, cure of, 360, 644; of the elbow, case of, 372; of the radio-carpal articulations, cases of, 654; of the femur, 659.
Dispensary, Philadelphia, reports from, 57, 217, 235, 443, 469, 615, 681.
Dissection, preservation of subjects for, 116.
Dissector, the London, 115.
Division of nervous fibres, 730.
Dropsy, lecture on, 110; periodical uterine, 419.
Dublin Dissector, notice of, 693.
Dysmenorrhœa, mercurials in, 163; treatment of, 172; stramonium in, 728.

E

Elephantiasis, case resembling, 229; tubercular, 814.
Emphysema, cases of idiopathic, 447, 765; of the stomach, 531.
Encephalon, on the functions of the, 740.
Encouragement to men of science, 648.
Endocarditis, Dr. Stillé's case of, 21; lecture on, 27; Dr. Pennock's case of, 165.
Enteritis, case of, 313.
Entozoaria, Dr. Horner's specimens of, 549.
Erysipelas, M. Velpeau on, 636.
Esophageal arrest of development, 432.
Exostosis, case of, 225.
Experiments, medical, 629, 820, 825.
Exploration, physical, remarks on, 121.
Eye, formation of a new, 507.
Eye-lids, formation of new, 432.
Eyes, case of apoplexy of, 356.

F

Fall, case of injury from, 402.
Fever, congestive, Dr. Buck on, 25; yellow, Dr. Rufz on, 37, 53, 69, 101, 117; continued, Dr. Shattuck on, 133, 140; typhoid, lecture on, 140; on the intermittent and remittent of Peru, 478; on the bilious remittent of Peru, 499; on the gastro-catarrhal of Peru, 499, 508; on the nervous of Peru, 509; on the exanthemata of Peru, 512; on the simple gastric of Peru, 516; on the pain of the back in intermittent, 641; case of complicated intermittent, 695.
Fistula in ano, on M. Roux's operation for, 562.
Fracture, ununited, Dr. McCook's operation for, 155.
Fractures, Mr. S. Cooper on, 205; on the mode of union of, 516.

G

Gangrene of the lungs, lecture on, 61; of the mouth, 575.
Gastritis, Mr. Ferrall on, 541.
Generation, case of arrest of development of the organs of, 701.
Gentiana, chirayita, 266.
Gerhard, Dr., clinical lectures by, on pneumonia, pneumothorax, endo and pericarditis, 27; on pneumonia and delirium tremens, 46; on pleurisy, and gangrene of the lungs, 61, on jaundice, tubercular meningitis, and laryngeal phthisis, 93; on dropsy, 110; on asthma and paralysis, 122; on acute rheumatism, typhoid fever, and phthisis, 140; on the mode of examining patients, 159; on diseases of the chest, 309, 325, 341, 357, 373, 389, 405, 421, 437, 533, 581, 613, 661, 757, 821.
Glanders in the human subject, 741.
Graduates of medicine in United States during 1840, 454.
Graefe, death of, 564.
Grate, Dr., Professional accumulations of, 484.
Grease of the horse, analogy of, with vaccine, 611.
Gross's pathological anatomy, bibliographical notice of, 76.

H

Heart, Dr. Pennock on the situation of, 213; on penetrating wounds of, 419; rupture of the right ventricle of the, 432; British report on the sounds of, 645, 828.
Hematemesis, cases of, 179, 180.
Hernia of the lung, 227; diaphragmatic, 240; new facts in relation to strangulated, 526; of the fallopian tube, 623; reduction of strangulated inguinal, 624; on the radial cure of inguinal, 660.
Hip, difficulty of diagnosis in injuries of, 146.
Hip-joint, Dr. Mütter on luxations of the, 805.
Hospitals, Wills', reports from, 43, 774.
Hydrocephalus, puncture for, 577.
Hydrophobia, cases of, 443.
Hydropneumothorax, case of, 329.
Hydrostatic test in infanticide, 836.
Hydrosudopathy, account of, 521.
Hymen, imperforate, cases of, 158.
Hysteria, Sir B. Brodie on, 100.
Hysteritis, Dr. Fleet's case of, 171.

I

Iliac artery, ligature of, 260.
Illegitimate, statistics of, 196.
Inflammation, on the mechanism of, 525.
Insane, Vermont asylum for the, 648; Dr. Dunglison's appeal for the, 681.
Insanity, the premonitory symptoms of, 686.
Iodide of potassium, salivation from, 228.
Iodine in cod-liver oil, 642.
Ipecacuanha, tasteless form of, 340.
Iritis, Mr. Walker on, 697; M. Ammon on, 746, 763.
Iron, hydrated peroxide of, 236.
Irritation, spinal case of 197.
Ischuria, renalis, cases of, 431, 669.

Itch, professor Cazenave on, 15; in adults and children, 642.

J

Jackson, professor Samuel's valedictory address, 232.
Jaundice, professor James Jackson on, 90; lecture on, 93.
Joints, on the treatment of injuries of, 464, 578; on diseases of the, 585; on subcutaneous wounds of the, 670.
Journals, Maryland Medical and Surgical, 44; Western, 109; spirit of the French medical, 198, 245.

K

Kidney, disease of, 548.

L

Lactate of iron, 643.
Lactucarium, 323.
Laryngeal phthisis, lecture on, 93.
Larynx, œdema of, 227.
Laudanum, cases of poisoning from, 556, 561.
Lead, oxide of, effect of on the gums, 366.
Leeches, the danger of using old, 642.
Lepoides, Dr. W. Harris on, 85.
Lithotripsy, Dr. Randolph's case of, 215.
Liver, Dr. Sinnickson's case of abscess of, 170; Dr. Hallowell's case of cancer of, 597; on cirrhosis of the, 656; varieties in the form and position of the, 692; fatty, 708.
Lumbrici in the hepatic ducts, 564.

M

Malingering, 249.
Mammary abscess, Mr. Ferrall on, 470.
Manufactures, the influence of, on children, 547.
May, Dr., introductory lecture by, 44.
Medulla oblongata, on a remarkable diminution of, 483; anatomy of the, 577.
Menorrhagia, cured by monesia, 441.
Meningitis, Dr. Hallowell's case of, 230; tubercular, Dr. Gerhard on, 293; Dr. Green's case of, 488.
Menstrual blood, on the injurious qualities of, 449; microscopic characters of the, 628.
Mercurial trembling, 608.
Menstruation recidiva, case of, 656; complete absence of, 658.
Mercury, bi-sulphuret, death from, 115; for coxalgia, 572.
Milk-sickness, 115; Dr. Buck on, 792.
Monesia, account of, 207; Dr. Nancrede on, 215; Dr. Burns' cases treated by, 517; cases treated by, 573.
Monstrosity, Dr. Stillé's case of, 138; cases of, 370, 612.
Mortality in the Prussian army, 436.
Motor and sensitive roots of nerves, experiments on, 450.
Moxas, new, 276.
Myopia, instrument for treatment of, 617.

N

Nails, on the curved form of, 367.
Neonati, pathology of, 292.

O

Odontoid process, luxation of the, 576, 553.
Oils, essential, on the adulteration of, 740.
Oponis spinosa, for rheumatism, 740.
Ophthalmia, Velpeau's lectures on, 32, 82, 98; the Belgian epidemic of, 431.
Ozœna, case of, 355.

P

Parrish, Dr., case of, 184; tributes of respect to, 185.
Pathology, surgical, contributions to, 376.
Peace, Dr. E., appointment of, 795.
Perforations of the lungs, Dr. Martins on, 348; of the œsophagus, 676.
Pericarditis, researches into, 689.
Peru, on the diseases of, 731, 752, 767, 778.
Pharynx, case of abscess of, 493; cases of disease of, 735.
Phlebitis, hepatica neonatorum, 660; case of, 807.
Phthisis, prussic acid in, 195.
Pisa, scientific meeting at, 186.
Plague, of Egypt, 528.
Pleurisy, lectures on, 61; pathological specimen of, 148.
Pneumonia, lectures on, 27, 46, 61; statistics of, 196, 621; case of, 677; on collapse in, 786.
Pneumothorax, lecture on, 27; cases of, 212.
Potassa, M. Trousseau on, 453.
Premature labour, case of, 610.
Prolapsus ani, cured by actual cautery, 404; new operation for, 653.
Prussic acid, therapeutical action of, 227.
Purchasing a practice, 694.
Pus, on secondary deposits of, 716.

Q

Quinine, on the adulteration of, 384; dumbness produced by, 468, 574, 644; on the salts of, 580; on the hydrocyanoferrate, 670.

R

Rare cases, necessity of studying, 817.
Remedies, effects and modes of application of, 384.
Respiratory organs, on anomalous affections of, 604.
Rhatany, in the treatment of fissure of the anus, 551.
Rheumatism, opium in acute, 173.
Rush, Professor Wm., introductory lecture by, 201.
Russian practice, 523; longevity, 756.

S

St. Petersburg, mortality of, 756.

Salivation, from hydriodate of potash, 668.
Salt, in consumption, 175.
Salt-Sulphur Springs, notice of Dr. Mütter's pamphlet on, 333.
Saratoga water, Dr. North on, 295.
Scarlatina, Dr. Sale on, 42; dropsy following, 84; in the West, 115; new treatment of, 466; cases of, 811.
Scirrhus of the stomach, Professor Miller's case of, 91.
Scrofula, Mr. Phillips on, 251, 267, 285, 301, 317.
Sea-scurvy, 276.
Secale cornutum, on the primary action of the, 737.
Serous secretions, 432.
Soot, in diseases of the bladder, 340.
Spermatic vein, case of rupture of, 352.
Spine, softening of, 212; diseases of posterior column of, 388.
Spleen, Dr. West's case of fracture of, 154.
Springs, Virginia, letters from, 485, 518.
Strabismus, on the cure of, 370, 594, 650, 760.
Stricture, case of cured by slippery elm bougies, 147.
Strychnine, in paralysis, 176; in neuralgia, 819.
Subperitoneal abscess, case of, 491.
Suffocation during intoxication, case of, 554.
Sugar, to protect iodide of iron from decomposition, 283.
Sulphates of copper and zinc, as emetics, 670.
Surgeon General of the Army, report from, 217.
Suspension of life, 707; on suicide from, 725.
Synovial membranes, dropsy of, treated by tartar emetic, 691.
Syphilis, in animals, 174; Arabic practice in, 228; Dr. Johnston on, 789.

T

Tæniæ expelled by pomegranate-root, 668.
Tapping, new instrument for, 707.
Tarsus, new plan for amputation through the, 620.
Tendons, crepitation of the sheaths of, 177.
Tennessee Medical Society, 695.
Testes, on the sympathy of, with the cerebellum, 383.
Testes, case of in the abdomen, 469; on the treatment of diseases of, by compression, 574; strangulation of the tunica vaginalis, 643.
Tetanus, new remedy for, 530; cases of, 743.
Thoughts on medicine, 740, 756, 772.
Throat, on peculiar affections of the, 671, 681.
Tornado, at Natchez, 486.
Tracheotomy in croup, Dr. W. P. Johnston on, 5; case of, 308.
Transactions of New York Medical Society, 333.
Transfusion, in uterine hæmorrhage, 704.
Truss, Dr. Fletcher's, 205.
Tubercles, of the testes, Dr. H. H. Smith on, 261; on the prevention of, 324.
Tubercular phthisis, Sir C. Scudamore on inhalations in, 333.
Tumour of the lower jaw, case of, 400.
Turnpenny, sketch of Dr., 365.
Tweedie's practical medicine, notices of, 709, 773.

U

Ulceration of the throat, fatal case of, 212; of the lung, case of, 596.
Ulcers, Sir B. Brodie on, 17; corrosive sublimate for, 368; cold water douche for, 387.
Urethra probe, Dr. Turner's, 26.
Urinary organs, on abscesses accompanying diseases of the, 651.
Uterine hæmorrhage, cases of, 436, 442; M. Gendrin on, 781, 795.
Uterus, Dr. Hamilton's case of diseased, 157; Dr. Walker's case of polypus of, 280; case of forcible removal of, 455; on hydatids of, 475; on retroversion of, 526; case of extirpation of, 546; operation for procidentia of, 601, case of rupture of, 616.
Uvula, on an affection of, 174.

V

Vaccine virus, preservative powers of the, 577.
Valves, adhesion of aortic, 452.
Varicose veins, cauterization for, 571; new treatment for, 620.
Variola, varioloid, and varicella, occurring in the same individual, 655.
Variolous pustules in the bladder, 564.
Veins, M. Amussat on the introduction of air into, 501.

W

Wounds, on the cure of without inflammation, 569; on sub-cutaneous, 629.

